# Main elements of current spine biomechanics research: model, installation and test data

**DOI:** 10.3389/fbioe.2025.1646046

**Published:** 2025-10-20

**Authors:** Bide Xu, Gang Li, Xueheng Sun, Simengge Yang, Ran Sun, Ying Gong, Mingzhi Song

**Affiliations:** ^1^ Institute of Translational Medicine, Jining Medical University, Rizhao, China; ^2^ Institute of Translational Medicine, Shanghai Jiao Tong University, Shanghai, China; ^3^ Department of Orthopaedics, Union Hospital, Tongji Medical College, Huazhong University of Science and Technology, Wuhan, China; ^4^ School of Rehabilitation Science, Shanghai University of Traditional Chinese Medicine, Shanghai, China; ^5^ Department of Orthopaedics, The Second Xiangya Hospital of Central South University, Changsha, China; ^6^ Department of Nursing, The First Affiliated Hospital of Dalian Medical University, Dalian, China; ^7^ Operating room, The First Affiliated Hospital of Dalian Medical University, Dalian, China; ^8^ Department of Orthopaedics, The First Affiliated Hospital of Dalian Medical University, Dalian, China; ^9^ Department of Orthopedics, Affiliated Hospital of Jining Medical University, Jining, China; ^10^ Department of Orthopaedic Surgery, Shanghai Ninth People’s Hospital, Shanghai Jiao Tong University School of Medicine, Shanghai, China

**Keywords:** spine biomechanics, experimental models, digital modeling, loading methods, measurement techniques

## Abstract

Biomechanical principles are crucial for spinal research, enabling precise analysis of spinal behavior under various conditions. Through quantitative analysis and simulation of mechanical information, spinal function can be optimized, posture improved, and injury risk reduced. However, current methodologies in spine biomechanics remain incomplete and challenging for researchers to apply effectively. This paper reviews recent advances in experimental models, loading methods, instrumentation, and test data used in spine biomechanics research, aiming to provide a reference for research design and future developments in the field. Two primary types of research objects are highlighted: measurement models and digital models. Common loading modes include dynamic, quasi-static, and static approaches. Various experimental setups, each with distinct characteristics, play essential roles in simulating motion and collecting data. The most frequently used biomechanical indicators are also discussed. In summary, this work redefines, categorizes, and synthesizes the typical research workflow in spinal biomechanics. It is foreseeable that more unified, accurate, and innovative studies will enhance the application of spinal biomechanics in rehabilitation, treatment, auxiliary materials, and clinical evaluation.

## Introduction

Biomechanics is a branch of biophysics that applies the principles and methods of mechanics to carry out quantitative research on mechanical problems in organisms. The early studies of anatomy and physicists laid the foundation for modern science and biomechanics. At first, people were obsessed with the study of bone properties. Andreas Vesalius is the first scholar who described the biomechanics of spinal structure in detail from the anatomical level. Giovanni Alfonso Borelli applied the principles of “rotational balance” and “translational balance” to the analysis of spine biomechanics (Pope, 2005). In 1680, he published *The Animal Movement*, which is considered the pioneering work of biomechanics. It introduced many valuable calculation methods for spinal biomechanics research. Additionally, Borelli was the first to recognize the importance of the intervertebral disc structure, which supports most of the spinal load (Borelli, 1989). Leonhard Euler found that the human spine like a cylinder bears the compression load through the study of digital model, which would lead to spinal instability (Gribbin, 2010; Sanan and Rengachary, 1996).

Spine biomechanics showed a vigorous development trend from the mid-19th century to the early 20th century (Oxland, 2015). At that time, the research of spine biomechanics mostly focused on the military and sports fields (Guillemin 1950; Hirt, 1955). In the mid-19th century, Hirsch, together with Nachemson, pioneered the direct measurement of spinal load. They started with cadaveric intervertebral discs and gradually extended it to *in vivo* measurement of human body (Nachemson and Morris, 1964; Nachemson, 1966). The effects of these methods were also verified in later studies (Sato et al., 1999; Wilke et al., 1999). Lissner studied spinal biomechanics in the early 1950s (Gurdjian et al., 1949; Hardy et al., 1958; Evans and Lissner, 1959). Then, he conducted a study on the effect of axial compression and lateral bending on lumbar disc herniation. This was also considered to be the first truly modern spinal biomechanical experiment. Lysell had also made a great contribution to the *in vitro* study of cervical motion and movement patterns (Lysell, 1969). He used fresh cadaveric cervical spine specimens (C2-T1), inserted four steel balls into each vertebral body, and measured the relative three-dimensional motion of each vertebral body by taking quantitative stereogram. In 28 specimens, he found that age or the degree of degeneration of tissue structure had little effect on the range of motion (ROM) (Lysell, 1969). In the following decades, with the development of modern science and technology, the spine biomechanics with animal models and human models as the main research materials had made continuous progress, and gradually refined the four elements needed for these research: experimental model, loading method, experimental installation, test data. In the middle of the 20th century, the emergence of computers provided the most important technical basis for the digital development of models. Subsequently, the rapid progress of biomechanical digital technology continued to update iteration, the emergence of a finite element model as the representative of the digital model. Brekelmans et al. introduced the finite element method into the field of orthopedics for the first time in 1972 (Brekelmans et al., 1972). Then Schultz, Belytschko and Ha-Kim tried to apply the engineering research method of finite element analysis technology to spine modeling (Belytschko et al., 1974; Hakim and King, 1979). After the 1970s, spinal biomechanics underwent significant development, with key contributions from Panjabi and White in understanding spinal stability and mechanical behavior (Panjabi et al., 1976). Panjabi proposed the theory of spinal stability, emphasizing the role of ligaments and the three-joint complex in maintaining spinal stability, which laid the foundation for understanding spinal injury and instability mechanisms. White and Panjabi’s book *Clinical Biomechanics of the Spine* provided a comprehensive theoretical framework for the mechanical behavior of the spine. With advancements in computer technology, finite element analysis was applied to spinal modeling and simulation, enabling more accurate representations of spinal dynamics, injury mechanisms, and treatment outcomes. In the 21st century, spinal biomechanics research further progressed with the rise of patient-specific modeling and multiscale modeling, incorporating CT, MRI, and other medical imaging technologies to create precise models based on individual differences. Additionally, the integration of artificial intelligence and machine learning has opened new possibilities for spinal surgery planning, rehabilitation, and pathological analysis, further advancing both clinical applications and research in spinal biomechanics.

However, as the field has expanded, the issue of lacking standardization has become more pronounced. The absence of universally accepted standards has resulted in considerable inconsistencies in experimental designs, data collection methods, and model simulations, leading to difficulty in comparing results across different studies. Additionally, the reproducibility and reliability of spinal biomechanical models are compromised, affecting the clinical translation of research findings. Therefore, we hope to provide more diversified design ideas and better element combination schemes for the research in this field.

## Experimental model

### Measurement model

The origin and development of spine biomechanics is based on the initial exploration of human body model. The experimental results obtained by using human model for functional research are more convincing. Both the most widely used animal models and the emerging finite element models are based on human models. All measurement models were summarized in Table 1.

**TABLE 1:** Summary of model types.

Category	Model type	Advantages	Disadvantages	Methods or tools of model processing	Test data	References
Measurement model	*Human*	High credibility, accuracy and complete structure	Limited by access difficulties, regional customs differences, funding and experimental conditions	Excluding bone diseases and deformities; preserving the ligaments (such as intertransverse, ligamentum flavum, and facet capsular ligaments), intervertebral discs and bony structures	ROM; neutral zone; strain; average displacement of disc; elastic zone stiffness; neutral zone stiffness; elasticity; euler angles; kinematic transformations; hysteresis area; intervertebral displacement; moment-angle plots; stiffness; disc space height; intravertebral angle; lumbar angle; intradiscal pressures; behavior analysis	Lea and Gerhardt (1995), Rao et al. (2002), Nayak et al. (2013), Wang et al. (2013b), Aghayev et al. (2014), Gonzalez-Blohm et al. (2014), Bell et al. (2016), Liu et al. (2016), Du et al. (2017), Lipscomb et al. (2017), La Barbera et al. (2018), Liebsch et al. (2018), Lindsey et al. (2018), Kim et al. (2019), Sawa et al. (2020)
	*Baboon*	Similar cervical spine structure	High cost, limited access, and strict ethical constraints			Sheng et al. (2010)
	*Large animals*	Similar spine traits	Elevated infection risk in pigs; high husbandry cost and stress susceptibility in sheep			Smit (2002), Pflugmacher et al. (2004), Hoogendoorn et al. (2008), Tai et al. (2008), Schwab et al. (2009), Cunningham et al. (2010), Gu et al. (2010), Patel et al. (2010), Roth et al. (2013), Ho et al. (2015), Li et al. (2015), Wang et al. (2019), Chow and Gregory (2023), Gullbrand et al. (2025)
	*Rabbit*	Similar lumbar spine structure	Small cervical dimensions and divergent mechanical responses vs. humans			Roth et al. (2013)
	*Rat and Mouse*	Highest maneuverability and controllability; the best choice of spinal cord injury	Small spinal size, limited load-bearing, and high surgical precision requirements			Sharif-Alhoseini et al. (2017), Danilov et al. (2020), Nishi et al. (2020)
Digital model	*FE model*	Flexible, repeatable, not affected by experimental environment and model deterioration	Lack of standardized procedures; CT fails to capture intervertebral disc data	CT; MRI; 3D structure reverse generation: Avizo, Amira, VG Studio, Mimics; analysis softwares: LUSAS, MSC Nastran, ANSYS, ABAQUS, LMS-Samtech, Algor, Femap/NX Nastran, HyperMesh, COMSOL Multiphysics, FEPG	ROM; strain; stress; intradiscal pressure; facet joint force; disc displacement; disc inclination angle and height; joint displacement; concentrated force; moment-intervertebral rotation proportion; facet load pressure; intradiscal pressure; spinal mobility; intervertebral disc; vertebral motion segment; displacement magnitude	Lipscomb et al. (2017), Elmasry et al. (2018), Huang et al. (2018), Zhang et al. (2018), Guan et al. (2019), Zhou et al. (2019), Ahuja et al. (2020), Correia et al. (2020), Fan and Guo (2020), Goertz et al. (2020), Guan et al. (2020), Jia et al. (2020), Ouyang et al. (2020), Wong et al. (2020), Fasser et al. (2021), Sun et al., 2022, Gribbin (2010), Saini et al. (2023), Stott and Driscoll (2023), Yu et al. (2024), Gullbrand et al. (2025), Lin et al. (2025)
	*Motion capture model*	Studying the function of spine and surrounding muscles during human movement	Limited accessibility to tissue-change data across spinal regions	Vicon, Sega Interactive, MAC, X-Ist, Filmbox, CHINGMU, Nokov	ROM; muscle activity state; gait analysis	Bourdon et al. (2017), Lee et al. (2019), Schmid et al. (2019), Shin and Yoo (2019)

Human models have been used in various research fields of spinal biomechanics, such as physiological characteristics of spine (Sawa et al., 2020), surgical prediction of spinal diseases (Chen et al., 2020), and surgical implants (Ludwig et al., 2000; Wang T. et al., 2013). The advantage of human body model lies in high credibility, high accuracy and complete structure. Therefore, when the model structure selected in the study is closer to the human’s, the reliability of the experimental results will be more convincing (Bozkus et al., 2001). But the application of human body model is more and more limited. For example, it is difficult to obtain the model, ethical Limitations, regional customs differences, funding and experimental conditions.

After many years of screening and trying, mammals have been the main species that can be used for spine biomechanics research, including monkeys, dogs, pigs, sheep, cattle, rabbits and mice. The advantages of animal models are abundant in quantity and easy to obtain. However, the spinal structure of animals is different from that of human beings. For example, TES-induced electric field strength strongly decreases from smaller to larger specimen with up to 100-fold differences across species (Alekseichuk et al., 2019). And the differences in weights, sizes, and characteristics can lead to significant inter-animal variation (Schimandle and Boden, 1994). Therefore, animal models should be selected according to the needs and characteristics of biomechanical research.

Primates have closely evolution relationship with human beings. Monkeys, orangutans, baboons and so on are available for selection in biomechanical research. For example, the spine structure of baboon, especially the cervical spine structure, is very similar to that of human (Sheng et al., 2010). Secondly, the vertebrae anatomical structure of large animals such as pigs, cattle, sheep and dogs in order of perissodactyla and artiodactyla is close to that of human beings. And the stress of spine in physiological state which loaded along the long axis is similar to that of human spine, so it can be the first choice for most spine biomechanical studies (Smit, 2002). Pigs were selected most in scoliosis correction studies (Schwab et al., 2009; Patel et al., 2010; Roth et al., 2013). The biomechanical properties of sheep spinal model are also outstanding, which is also a good choice for spinal biomechanical research (Hoogendoorn et al., 2008; Wang et al., 2019). In addition, some studies have found that the anatomical structure of rabbit and human lumbar spine is similar, and there is enough iliac bone as a support, which is suitable for some studies with low load and stress requirements (Roth et al., 2013). Although the size of rats is small, they have the highest maneuverability and controllability, which is the best choice for the study of spinal cord injury (Sharif-Alhoseini et al., 2017; Danilov et al., 2020; Nishi et al., 2020).

In addition to the above common animal models, some new species have also gradually attracted people’s attention. Kangaroo is an upright animal, which makes the mechanical properties of its spine more like the spine of human beings (Balasubramanian et al., 2016). Some scholars use Japanese small game fowls to study cervical kyphosis (Shimizu et al., 2005). In addition, researchers are also committed to developing new model species through artificial intervention, such as Ao et al., who successfully established a bipedal standing mouse model for the study of intervertebral disc degeneration (Ao et al., 2019).

### Digital model

In recent years, due to the rapid development of computer technology, a variety of digital spine research models emerge in endlessly, among which the finite element model and motion capture model are the most widely used. All digital models were summarized in Table 1.

Three-dimensional finite element analysis technology uses the principle of mathematical approximation to simulate the real physical system by setting geometry and load conditions. The birth of finite element model depended on the continuous progress of digital model. The early digital model come from three-dimensional reconstruction technology, such as VOXEL-MAN system (Schiemann et al., 2000), but which has great limitations in the use value and application scope (Jager, 1996). Until the mid-20th century, the finite element model began to rise and was gradually used in various fields of medicine, and emerged in the field of spine biomechanics (Tien and Huston, 1985; Zhai et al., 2019). Researchers use the detection instrument to scan the spine of subjects, and get the required finite element model after software processing. Finite element models of spine often appear in four forms, including whole spine model, local segment model, normal model and pathological model (Liu et al., 2017). Among them, the whole spine model is mostly simplified, which is composed of several segments and the interrelation among the segments (Lalonde et al., 2013). Common reconstruction software could reverse generate 3D model from scanning data, such as Myrian XP-Liver, HexaUnion3D and Mimics. The establishment methods of gender spinal models have also made initial attempts. (Hindman et al., 2020; Stott and Driscoll, 2024). Preparation of personalized model using 3D printing from MRI, CT data is a technique which is cost effective, time efficient, high fidelity, biomechanically accurate and enables proof of concept for simulating finite element models of the lumbar spine (Bohl et al., 2020; Dukkipati and Driscoll, 2025). Moreover, 3D printing technology can make the new type of model, which contains the advantages of both human model and digital model (Clifton et al., 2019). Finite element model is flexible, repeatable, and not affected by experimental environment and model deterioration. Therefore, it is often used in some destructive experiments to collect model change data at multiple time points, such as impact test (Huang et al., 2018; Correia et al., 2020). At the same time, the construction of finite element model has strong pertinence and can realize individual service and research, such as stress state of spinal tissue and artificial implant (Elmasry et al., 2018; Huang et al., 2018; Zhang et al., 2018; Zhou et al., 2019; Guan et al., 2020; Jia et al., 2020; Ouyang et al., 2020; Wong et al., 2020). In addition, traditional finite element models of the spine require laborious segmentation of medical images and extensive computation, but AI methods now automate and accelerate this process. For example, deep learning can generate patient-specific finite element models from MRI scans by creating synthetic CT images and automatically segmenting vertebrae and discs, achieving high geometric accuracy without exposing patients to radiation. This individualized modeling overcomes the time-consuming manual workflows and ensures that simulations reflect a specific patient’s anatomy and tissue properties.

The establishment of motion capture model is different from the finite element model. It is to set up a tracker in the key parts of the moving object, then by monitoring the position of the tracker, the three-dimensional coordinate data can be obtained after computer processing. When the data is recognized by computer, the motion capture model is formed in the software, which can be applied to gait analysis, biomechanics, ergonomics and other fields. At present, a variety of commercial motion capture devices have been developed, among which the optical motion capture system is widely used in the field of spine biomechanics. The advantage of motion capture model is not only its authenticity and non-destructive, but also its real-time monitoring, which is convenient for dynamic research. Combined with AI technology, it allows for the estimation of 3D body shape and spinal kinematic postures with just one camera (Ghezelbash et al., 2024). Therefore, it is widely used in the field of spinal biomechanics, such as sports and rehabilitation exercise.

### Model preprocessing

After selecting the appropriate model type, we should choose a reasonable method to preprocess the model. The purpose is to remove the redundant tissue in the model to optimize the experimental scheme, so as to obtain the experimental data more directly.

Firstly, one or more spinal segments were selected according to the needs of the study. Panjabi et al. have pointed out that the functional spinal unit (FSU), defined as two adjacent vertebrae with interconnecting connective tissue, was the basic anatomic unit of study requires dividing the specimen into interest segment (Panjabi et al., 1976). The more FSUs involved in the study, the more factors need to be considered, such as the physiological curvature of the spine and the interaction between the structures, which will increase the difficulty of the experiment and affect the accuracy of the results. Secondly, muscle and fat were removed from the study subjects for pre-processing. It should be noted that ligaments, intervertebral disc and bony structure of spine should be retained (Chazal et al., 1985; Pintar et al., 1992; Han et al., 2012; Bell et al., 2016; Lipscomb et al., 2017; La Barbera et al., 2018). After pretreatment, the spinal structure is relatively complete, which can effectively separate the spinal part from other parts. It is worth noting that the influence of ribs on the stability of thoracic vertebrae model should be fully considered when the thoracic vertebrae model is pretreated. A large number of studies have confirmed that the thoracic cage could provide passive stability for the spine to a certain extent (Sham et al., 2005; Ignasiak et al., 2016; Liebsch et al., 2018). Thirdly, in order to fully ensure the representativeness of the model selected, it is necessary to use detection instruments to assist researchers in excluding unqualified model individuals. Before most spine biomechanical studies, X-ray examination is needed for the subjects to exclude bone diseases and deformities of the model (Xue et al., 2013; Li et al., 2015; Sturges et al., 2016; Liebsch et al., 2018). And bone mineral density examination instrument can also be used to check osteoporosis of the model (Nayak et al., 2013; Aghayev et al., 2014). Finally, animal models and human models are *in vitro* tissue, and the structure of soft tissues such as ligaments will change with time. Therefore, formal experiments should be started after 2–5 pre-experiments to reduce the impact caused by the viscoelastic effect of the model itself (Wilke et al., 1998).

## Experimental loading mode

### Dynamic experiment

There are two kinds of dynamic tests. One is the destructive tests with the change of load rate or deformation rate with time, such as fatigue test, impact test and dynamic simulation test of products. The other one is the non-destructive test to study the properties of materials by periodic stress or deformation. Dynamic loading method is mostly used for the study of spinal joint damage (Liu et al., 1983; Tsai et al., 1998) and strength of artificial implants (Burval et al., 2007).

### Quasi static experiment

Quasi static process is the sum of all the states that the system experiences when it changes from one equilibrium state to another. Quasi static loading is the most common loading method in spine biomechanics. In the process of slow loading, the related structures of the spine will not be damaged, which can better display the functions of the spine and related structures in daily state. The research of the complex mechanical behavior of a certain segment of the spine (Liebsch et al., 2018; Yamamoto et al., 2022) and the stability of artificial implants after spinal surgery (Pflugmacher et al., 2004; Gu et al., 2010; Aghayev et al., 2014; Gonzalez-Blohm et al., 2014) are the typical examples.

### Static experiment

Static test is a kind of strength test and different with dynamic test. Its characteristic is small load, slow deformation speed and short measuring time. Tensile test and hardness test are often adopted static test. In the research of spine biomechanics, the pure static loading is rare, and the static loading and quasi-static loading are usually carried out alternately.

## Experimental installation

### Installation for physical model testing

The main function of the platform is to drive the model to make various preset movements, such as flexion, extension, lateral bending and rotation. The applicability and operability of the test platform will directly affect the effect of the spinal model to complete the related movements. Installation of spine biomechanics for physical model testing was summarized in Table 2.

**TABLE 2:** Summary of installation of spine biomechanics.

Category	Major installation	Matching installation	Effects	Advantages	References
Testing machines	MTS system; TestResources testing machine; Instron testing machine; Stewart platform	Cable and pulley tools; 6-DOF spine simulator; Fixing materials (polymethylmethacrylate; the mixture of glass fiber resin and body filler; metal parts)	Model fixation and preset motion simulation	High operation precision, strong flexibility, and a wide range of uses	Kandziora et al. (2002), Rao et al. (2002), Gillespie and Dickey (2004), Pflugmacher et al. (2004), Tai et al. (2008), Cunningham et al. (2010), Nayak et al. (2013), Wang et al. (2013a), Aghayev et al. (2014), Gonzalez-Blohm et al. (2014), Ho et al. (2015), Sturges et al. (2016), Wilke et al. (2016), Du et al. (2017), Lipscomb et al. (2017), Liebsch et al. (2018), Lindsey et al. (2018)
Imaging instruments	X-ray; CT; MRI	—	Exclusion of spinal deformities; provision of raw data for finite element analysis	X-ray: simple, widely used; CT: primary source for finite element model data; MRI: detailed visualization, especially of soft tissues	Nayak et al. (2013), Wang et al. (2013a), Wilke et al. (2016), Fournely et al. (2018), Liebsch et al. (2018), Lindsey et al. (2018), Arnold et al. (2019), Kim et al. (2019), Sawa et al. (2020)
Motion capture system	Vicon optical motion capture system; Optotrak Certus motion capture system	Surface electromyography system; force platform	Acquisition of 3D motion trajectories	ROM calculation and kinematic analysis of spine–muscle dynamics during movement	Lea and Gerhardt (1995), Pflugmacher et al. (2004), Cunningham et al. (2010), Nayak et al. (2013), Wang et al. (2013a), Wilke et al. (2016), Bourdon et al. (2017), Liebsch et al. (2018), Lindsey et al. (2018), Arnold et al. (2019), Kim et al. (2019), Lee et al. (2019), Schmid et al. (2019), Shin and Yoo (2019), Sawa et al. (2020)
Other measuring instruments	Strain gauge; non-contact optical 3D strain measuring system	Strain analysis system	Surface forces measurement of spine and implants	Assessment of stress concentration and distribution in both spine and implants	Ho et al. (2015), La Barbera et al. (2018), Song et al. (2021) Lin et al. (2025)

The most widely used testing platform in spine biomechanics research is the commercialized testing machines. And MTS system company’s testing machine is the most commonly used testing platform characterized by its high operation precision and strong flexibility, such as 858 Mini bionix type II MTS testing machine. This kind of platform had been used in the research of the stability of sacroiliac joint, the related performance test of intervertebral fusion cage and other research of spine biomechanics (Aghayev et al., 2014; Gonzalez-Blohm et al., 2014; Liu et al., 2016; Lindsey et al., 2018). Du et al. also used the MTS system of Model 608.33. in the research on the influence of intervertebral cage height on lumbar spine (Du et al., 2017), which has stable testing effect and has become the preferred platform for most spinal biomechanical studies (Pflugmacher et al., 2004; Tai et al., 2008; Cunningham et al., 2010). Besides, TestResources testing machine (Nayak et al., 2013), Instron testing machine (Ho et al., 2015; Lipscomb et al., 2017) and Stewart platform (Gillespie and Dickey, 2004) can also be good choices for spine biomechanical research.

Although the commercialized testing machine is a mature experimental platform, its function is still limited. Therefore, in the research with special needs, some auxiliary devices used with the testing machine also play a vital role. The most common auxiliary device is the cable and pulley tools (Kandziora et al., 2002; Rao et al., 2002; Pflugmacher et al., 2004; Gu et al., 2010; Nayak et al., 2013). The testing machine is used to drive the cable and pulley tools to apply pure bending moment, so as to induce bending, extension, left and right lateral bending, left and right axial rotation and other motion states. This system can make the model better simulate various actions under physiological state, so it is widely used. Gillespie et al. had used the improved Stewart platform to assist the model to produce different actions, which also achieved the ideal effect (Gillespie and Dickey, 2004). In addition, some researchers had developed a special six degrees of freedom (6-DOF) spine simulator, which could be used with the testing machine to simulate different spinal movements more conveniently and efficiently, and was expected to become the ideal measurement platform in future spinal biomechanics research (Xue et al., 2013).

After choosing the most suitable testing machine, the next key problem is how to fix the model on the test platform. There are two key points in the selection of fixation method: the fixation device should be able to adapt to the tested models; and under the condition of meeting the experimental requirements, the fixation device should give the model enough stability to prevent the model from fretting during the experiment and affect the research results. These two requirements make the fixture must have strong adaptability and stability.

In the past, a variety of materials have been used to fix the spinal model, the most commonly used are resin materials and metal materials. Resin materials have strong shaping, simple fabrication and high fit with the model, but poor fixation performance. Although the manufacturing process of metal materials is complex and the plasticity is poor, their firmness is often better than other common materials. Metal materials are generally used in the form of fixtures or tooling. Many studies choose metal parts combined with resin materials to fix the model. In Barbera’s study, for exploring the application effect of anterior interbody fusion cage and auxiliary rod, they embed the head end and tail end of the spine with polymethylmethacrylate (PMMA), and then PMMA is connected with the endplate structure at both ends of the model through screws, so as to achieve better reinforcement effect (La Barbera et al., 2018). The similar fixation method was used by Liebsch, Wilke and others (Pflugmacher et al., 2004; Nayak et al., 2013; Wang M. et al., 2013; Wilke et al., 2016; Liebsch et al., 2018). In addition to PMMA, Aghayev and Sabrina used the mixture of glass fiber resin and body filler to fix the spinal model in the *in vitro* study of spinal biomechanics (Aghayev et al., 2014; Gonzalez-Blohm et al., 2014).

Although a variety of fixation devices and methods for spinal models have been reported, there is still no unified fixation scheme. The reason may be related to the variety of spinal models and individual differences, which easily leads to the fixation device cannot be applied to all spinal biomechanical experiments. From the current research situation, resin material combined with metal parts for model fixation is a method with the strongest applicability. Moreover, other special fixation devices have complex process and small scope of application, but its advantages of reusability can also ensure the stability of the fixation and improve the experimental efficiency.

Clinically, physical model testing combined with cadaveric experiments provides biomechanical references for precision treatment in spinal surgery. As demonstrated by O'Hehir et al. using cadaveric biomechanical techniques, they proved that tethers can generally delay the onset of proximal junctional kyphosis and reduce angular changes (O'Hehir et al., 2024).Rudy et al. used either 2 or 4 screws and 2 or 4 rods for pelvic fixation on cadaveric models (Rudy et al., 2025). This approach showed no significant changes in strain in proximal connecting screws or rods in the cadavers, demonstrating that robust pelvic fixation can prevent distal failure and does not impose harmful effects on the proximal junction.

### Installation for finite element model testing

X-ray is mainly used to exclude bone abnormality and deformity of spinal model before finite element study (Zhang et al., 2018). CT is often used for spiral scanning and tomography of the spine in research. The thinner the slice, the finer the three-dimensional image, which provides accurate original data for the later finite element analysis of neck and chest. At the same time, CT scan can also check whether the spinal model is normal (Elmasry et al., 2018; Guan et al., 2019; Ahuja et al., 2020). The data obtained from CT scanning can be used for 3D reconstruction of finite element model. MRI scans are the preferred choice for soft tissue reconstruction, but they also have issues such as high cost, difficulty in identifying tissue structures, and an immature unified coordinate system. Therefore, in the future, we should try to use different sequences of MRI scanning data to establish intervertebral disc, muscle, ligament and other structures, and optimize the calculation method, in order to provide more help for the soft tissue research in spine biomechanics. Installation of spine biomechanics for finite element model testing were summarized in Table 2.

The commonly used finite element analysis softwares for 3D structure reverse generation are Avizo, Amira, VG Studio, Mimics, etc. And common finite element analysis softwares include LUSAS, MSC Nastran, ANSYS, ABAQUS, LMS-Samtech, Algor, Femap/NX Nastran, HyperMesh, COMSOL Multiphysics, FEPG, etc.

Currently, the application of finite element modeling in spinal research is advancing toward personalization, complex load simulation, and multi-scale integration. The development of multi-scale modeling urgently requires establishing reliable connections across tissue-cell-molecular levels to elucidate the mechanisms by which mechanical loading influences cellular metabolism (e.g., inflammatory responses, matrix degradation) (Vergroesen et al., 2015; Cheng et al., 2018). Furthermore, existing models are predominantly limited to quasi-static analyses. Future efforts should integrate dynamic complex loads (e.g., combined motions, high-velocity impacts) and leverage artificial intelligence algorithms to optimize real-time response predictions (Li et al., 2020). Landinez et al. have developed a patient-specific spine digital twin technology without requiring full 3D reconstruction (Landinez et al., 2025). This approach, based on conventional X-ray image segmentation algorithms and mathematical models of disc degeneration, enables quantitative calculation of intervertebral disc strain through finite element analysis. Multi-physics coupling also represents a critical future direction. By integrating electrophysiological signals to predict nerve root compression (e.g., triggering automatic pain simulation when displacement exceeds 1.2 mm), this strategy can ultimately realize the construction of nerve root compression models for degenerative spinal pathologies (Sun et al., 2025).

### Installation for motion capture model testing

The optical marker-based motion capture system is the gold standard for spinal biomechanics research, enabling precise quantification of kinematic parameters through non-invasive skin markers. The core components of the testing apparatus include an optical tracking system (e.g., infrared camera arrays), passive markers or active sensors, a data processing unit, and specialized analysis software (e.g., Vicon, Optotrak Certus, MotionAnalysis). The Plug-in-Gait marker set is the most common configuration, typically combined with lumbar marker optimization (e.g., replacing T10 with T12) to enhance accuracy (Hays et al., 2021). Multi-segment analysis requires dense marker strategies, such as the mesh grid protocol (8-row × 5-column layout) or anatomical landmark-derived models (Eldar, 2020).

During data acquisition, software (e.g., Vicon Nexus, Visual3D) converts marker coordinates into skeletal models to calculate kinematic parameters, including the ROM, angular velocity, acceleration, and instantaneous center of rotation (ICR) between spinal segments. Synchronization with surface electromyography or inertial measurement units allows simultaneous recording of paraspinal muscle activation timing and intensity, facilitating biomechanical analysis of “muscle-skeletal” synergistic motion. Studies indicate that the lumbar spine is a primary focus (56.25% of studies) due to its high mobility and load-bearing function, whereas cervical spine research is limited (Hesby et al., 2019). Common test activities include walking (37.5%), standing (25%), and flexion-extension ROM (26.79%), with fewer studies on sports or occupational movements (Glover et al., 2021). Multi-segment protocols require specific designs, such as adding thoracic markers (T3/T4) to track T10 vertebral motion (Rahmatalla et al., 2008).

Analytical methods focus on kinematic and kinetic parameters. Angular parameters (flexion/extension, lateral bending, axial rotation) are reported in 71.43% of studies, with flexion-extension being the most common (Mousavi et al., 2018). Kinetic parameters are involved in only 10.71% of studies and require estimation via musculoskeletal models (Dehghan and Arjmand, 2024). Mathematical models significantly influence results, such as coordinate system establishment methods and rotation sequence selection (Crawford et al., 1996; Anderst and Aucie, 2017). Direct vertebral motion measurement is only achievable through bone-pin marker techniques (MacWilliams et al., 2013). Recently, AI-assisted single-camera, markerless motion capture technology has enabled low-cost kinematic parameter extraction via deep learning and provide reliable gait parameters for certain applications, but large-scale training datas for generalizability are still necessary (Dinh et al., 2025; Usami et al., 2025). Commonly used motion capture software includes MotionAnalysis, Vicon, Sega Interactive, MAC, X-Ist, Filmbox, CHINGMU, and Nokov. AI and machine learning applications are transforming how spinal kinematics and kinetics are captured, moving beyond the confines of laboratory instrumentation. Traditional motion capture systems provide high fidelity data but are expensive, require dedicated lab spaces, and rely on skilled operators–factors that limit their accessibility. AI-driven approaches are overcoming these barriers by leveraging computer vision and wearable sensors for spinal motion tracking. One major advance is the use of markerless pose estimation. Modern deep learning algorithms can reconstruct 3D body shape and spinal posture from ordinary video, even a single camera feed. These methods, combining computer vision with learned biomechanical models, achieve accurate estimation of spinal alignment and joint angles without any wearable markers. As a result, capturing spine movements has become more practical and affordable. A single smartphone or camera can now yield rich kinematic data, making biomechanical analysis “field-ready” and not restricted to motion labs.

## Test data

In the field of spine biomechanics, researchers rely on many types of experimental measurements to characterize how the spine behaves under various conditions. In summary, test data serve as the foundation for advancing both basic and applied research in spinal biomechanics. On one level, they provide benchmarks for validating computational models, ensuring that finite element analyses and simulations remain consistent with physiological reality. On another, they act as diagnostic indicators of spinal stability and pathology, allowing researchers to identify abnormal loading patterns, instability, or degeneration. Test data are also indispensable for the design and evaluation of implants, as they reveal how different devices redistribute stresses, alter kinematics, or affect adjacent segments. This section focuses on the data types most frequently reported in prior studies and highlights their special significance to biomechanical analysis and clinical outcomes.

### General data

ROM is the most important parameter in spinal kinematics. ROM describes the sum of neutral zone(NZ) and elastic zone(EZ) in one direction of motion. By comparing the ROM data, we can study the stability of the spinal model joint, the factors hindering joint activity and therapeutic effect, such as the evaluation of the curative effect of anterior cervical surgery (Cunningham et al., 2010), the biomechanical comparison of artificial implants (Pflugmacher et al., 2004), the research of joint stability (Lindsey et al., 2018). In the past, the ROM measurement methods were completed by visual measurement or a measuring tool (Lea and Gerhardt, 1995). In recent years, a large number of spine biomechanics research use high-speed dynamic tracking system to obtain real-time ROM data(Nayak et al., 2013; Wang M. et al., 2013; Wilke et al., 2016; Liebsch et al., 2018; Lindsey et al., 2018; Arnold et al., 2019; Kim et al., 2019; Sawa et al., 2020). For example, Bell et al. used Vicon optical motion capture system in the biomechanical study of cervical spine (Bell et al., 2016). In the finite element model, ROM can be directly obtained by computer software measurement, and the results are more accurate (Sun et al., 2022; Gribbin, 2010; Stott and Driscoll, 2023; Gullbrand et al., 2025; Lin et al., 2025).

Wilke et al. proposed in the study published in 1998 that the NZ refers to the angle difference between the two stages of exercise at zero load. The deformation from the end of the NZ to the maximum load point is defined as the EZ (Wilke et al., 1998). The main manifestation of NZ is the activity of ligaments around the spine and other soft tissue under relaxation, while EZ is the activity of ligaments under tension. Therefore, NZ and EZ can show the movement ability and stability of spine. NZ and EZ are very common in the experimental measurement of spinal biomechanics, especially in the evaluation of spinal joint stability (Pflugmacher et al., 2004; Gonzalez-Blohm et al., 2014; Sawa et al., 2020; Lin et al., 2025). The concept of NZ stiffness was also mentioned in Wilke et al. The NZ stiffness was defined as the quotient of load and deformation (Wilke et al., 1998; Aghayev et al., 2014).

Strain can be captured by strain gauge and computer software measurement. Resistance strain gauges can sense mechanical deformation on the surface of an object and reflect the magnitude of the strain force through changes in resistance values. In Ho’s study on the efficacy of minimally invasive lumbar decompression, strain gauges were placed on the surface of L3 and L4 vertebrae to measure the strain of different segments, which can be used to study the stability of lumbar spine after laminectomy (Ho et al., 2015). Song et al. inferred the values of the stress concentration areas by obtaining the strain values of the internal fixation system (Song et al., 2021). Lin et al. achieved high-precision multidimensional strain analysis in cervical spine specimen studies by improving specimen preparation and applying a non-contact optical 3D strain measuring system, revealing the strain concentration of soft tissues under tensile loading and the consistency of deformation of the C4-C7 segments (Lin et al., 2025). In addition, the measurement of stress-strain under the finite element model is not limited by the artificial implant site and the number of stress distribution measurement points, enabling individualized service and research (Sun et al., 2022; Kassab-Bachi et al., 2023; Gullbrand et al., 2025).

### Other data

In addition to common indicators, there are many other indicators also used to catering to some special needs of spine biomechanics research. In addition to common indicators, many specialized measures are used in spine biomechanics research to address specific needs. For example, intervertebral disc height, intervertebral angle, and lordosis angle have been measured to evaluate the biomechanical stability of a cap-type cervical interbody fusion cage (Gu et al., 2010). The helical axis of motion (HAM) is another important parameter. Its position and orientation in the cervical spine were first identified in 1993 (Milne, 1993). Energy loss has also been utilized as an evaluation metric for spinal stability after injury, with *in vitro* studies reporting satisfactory results (Doulgeris et al., 2013; Gonzalez-Blohm et al., 2014). Another kinematic indicator is the instant center of rotation (ICR), defined for each motion segment as the point where its axis of rotation intersects the inferior vertebra’s sagittal plane (Wawrose et al., 2021). Leveraging this concept, a follower load technique can apply compressive force through each segment’s ICR to overcome limitations in axial loading (Patwardhan et al., 1999). Furthermore, anatomical factors such as intervertebral disc height and bone mineral have been used to gauge the physiological status of spinal models (Nayak et al., 2013; Aghayev et al., 2014; Kim et al., 2019; Sawa et al., 2020).

Besides the ROM and the strain force on the surface of the object, the finite element analysis can also make more precise measurement (Elmasry et al., 2018; Zhang et al., 2018). For example, in the impact test, the finite element analysis can calculate the displacement of the vertebral body or the center of gravity through the established coordinate system to understand the structural changes after the impact (Correia et al., 2020). Finite element analysis also facilitates forecast of internal spinal responses: measuring intradiscal pressure (IDP) can demonstrate the effects of decompression procedures (Zhou et al., 2019). With the aid of computer technology, the center of rotation can also be measured and used in the finite element analysis for dynamic experiments (Milne, 1993; Jager, 1996). In the motion capture model, in addition to the commonly used ROM, the motion trajectory of the subject in the spatial coordinate system was monitored, and the data of muscle activity state and gait analysis were obtained through the additional device (Bourdon et al., 2017; Lee et al., 2019; Schmid et al., 2019; Shin and Yoo, 2019).

Despite methodological limitations and variability across studies, continuous improvements in measurement techniques, such as high-resolution imaging, telemetric sensors, and motion capture, are enhancing the reliability and clinical relevance of biomechanical datasets. Collectively, these data not only deepen our understanding of spinal biomechanics but also form the critical link between experimental research, computational modeling, implant innovation, and patient-centered care.

## Discussion

**FIGURE 1:**
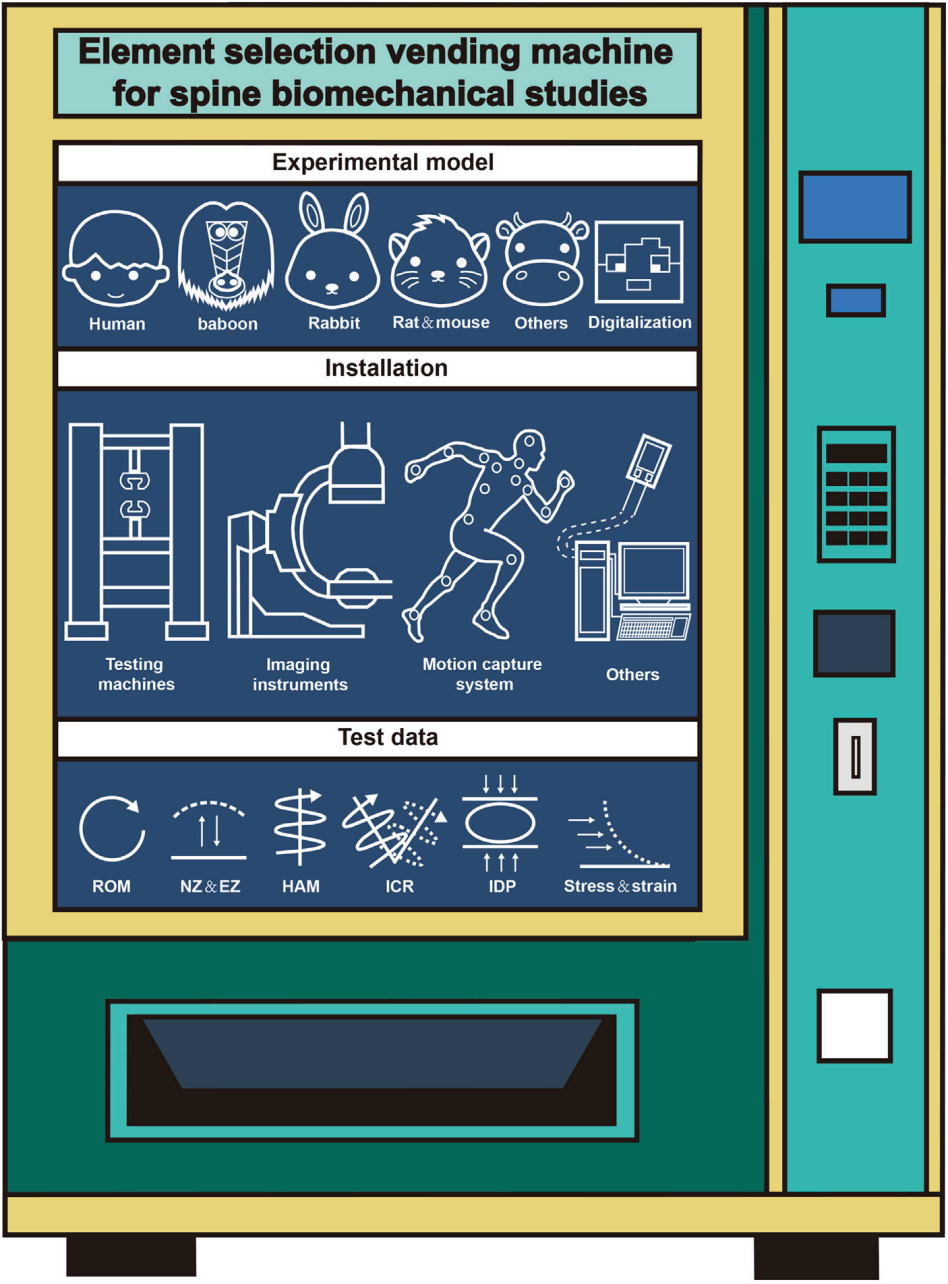
Selection of elements for spine biomechanics study.

Spinal biomechanics, as an interdisciplinary field, investigates the mechanical behavior of the spine through the integration of anatomy, engineering, and computational science. While significant advancements have been made over the past century, particularly with the rise of digital technologies like finite element analysis and motion capture, the field grapples with persistent challenges. Foremost among these is the lack of standardized experimental protocols and unified research guidelines. This absence leads to inconsistencies in model selection, experimental design, data acquisition, and analysis, hindering result comparability, reproducibility, and ultimately, the clinical translation of findings. This review has systematically reclassified and summarized the fundamental elements of spinal biomechanics research including experimental models, installations, and test data. Our goal of providing clearer guidance and diversified design frameworks for researchers navigating these complexities. Here, we redivided the research process of literature, reclassified and summarized systematically according to certain methods, mainly including experimental model, installation and test data (Figure 1). The ultimate goal is to provide a systematic theoretical framework for researchers of spinal biomechanics.

Three primary types of models commonly used in spinal biomechanics research are identified: human models, animal models, and digital models. Human models are highly valued for their anatomical accuracy and physiological relevance, making them the gold standard for studying spinal behavior. However, their use is often restricted due to ethical concerns, logistical challenges, and limited availability. Animal models, such as those based on primates, pigs, and rabbits, offer a practical alternative but present difficulties in terms of anatomical differences that may limit their direct applicability to human spinal mechanics.

Digital models, particularly finite element models, have become essential tools in the field, providing flexibility, repeatability, and the ability to simulate a wide range of loading conditions and spinal pathologies. While these models are increasingly popular due to their ability to provide personalized simulations, challenges remain in achieving the necessary standardization for their construction and validation. The development of digital models, including the integration of AI and machine learning, holds great promise for enhancing the accuracy and efficiency of spinal biomechanics research by automating model generation and analysis. In terms of installation, the review emphasizes the importance of high-precision testing machines such as MTS systems, instron testing machine and stewart platform. These platforms are used to simulate various spinal movements, such as flexion, extension, and rotation, under different loading conditions. The use of imaging technologies like X-ray, CT, and MRI scans is crucial for obtaining baseline data for model construction and validation, while motion capture systems and strain gauges are vital for capturing real-time kinematic data and stress distribution. In addition, the integration of AI and machine learning in testing has pushed forward the development of more efficient and accessible methods for data collection.

Test data are used to validate computational models and to understand the mechanical behavior of the spine under different conditions. This review identifies several key types of data frequently used in spinal biomechanics research, including ROM, strain, stress, and IDP. In addition, some other data were also mentioned such as HAM, ICR, intervertebral disc height, lordosis angle, and muscle activity, which are increasingly used to assess spinal stability and the effects of specific interventions. The use of finite element analysis allows for more precise measurements of internal spinal responses, such as the effect of decompression procedures on intradiscal pressure.

There are still some issues that researchers should pay attention to when designing spinal biomechanics research:

i. Although computer simulation experiment has become an important part in the field of spine biomechanics, the combined use of digital model and measurement model is expected to be more scientific and accurate. The combination can realize the mutual verification and complementary advantages. Before the mechanical testing of medical devices, finite element modeling can be used to optimize the design and predict performance. Following the evaluation of digital model, the function, histology and biomechanical characteristics of the device can be evaluated with use of measurement model (Goel et al., 2006).
ii. Although spinal biomechanics research has established a certain framework, spinal models still face issues such as large volume, inconvenient experimental operations, and the need for further improvement in data accuracy. Therefore, selecting the most representative model during the research preparation phase is a critical task:
  a. For measurement models, pre-processing operations are a crucial step. Excessively reducing the amount of tissue around the spine may lead to decreased structural integrity, compromised spinal stability, and inaccurate experimental results (Bell et al., 2018; Arnold et al., 2019).
  b. When using digital models in research, certain special models are difficult to obtain conventional experimental data for, such as burst fracture models.
  c. Additionally, due to the iatrogenic damage caused by surgery to the vertebral body, parameter measurements for surgical models cannot be standardized (Elmasry et al., 2018).
  d. Establishing uniform and appropriate operational standards for each part of the finite element model is also an urgent issue that needs to be addressed.
iii. The fusion of AI, machine learning, and multiscale modeling represents the next frontier in spinal biomechanics, offering the potential to enhance the precision of simulations and improve the effectiveness of treatments for spinal disorders. However, achieving these advancements will require continued efforts to address the existing challenges related to model validation, data consistency, and clinical translation.
iv. The purpose of this review is to summarize the experimental elements in spinal biomechanics research and provide researchers with standardized experimental design guidelines. However, it lacks the application of systematic review methodologies (such as PRISMA). This review provides an overview of the overall biomechanics research plan, but further discussion is needed on the design of plans for specific topics, such as a more in-depth exploration of spinal *in vitro* testing or computer simulations, to provide more targeted and valuable insights.
